# Northern Hemisphere Stationary Waves in a Changing Climate

**DOI:** 10.1007/s40641-019-00147-6

**Published:** 2019-11-21

**Authors:** Robert C. J. Wills, Rachel H. White, Xavier J. Levine

**Affiliations:** 1 University of Washington, Seattle, WA USA; 2 Barcelona Supercomputing Center, Barcelona, Spain

**Keywords:** Stationary waves, Climate change, Rossby waves, Climate dynamics, Atmospheric general circulation

## Abstract

**Purpose of Review:**

Stationary waves are planetary-scale longitudinal variations in the time-averaged atmospheric circulation. Here, we consider the projected response of Northern Hemisphere stationary waves to climate change in winter and summer. We discuss how the response varies across different metrics, identify robust responses, and review proposed mechanisms.

**Recent Findings:**

Climate models project shifts in the prevailing wind patterns, with corresponding impacts on regional precipitation, temperature, and extreme events. Recent work has improved our understanding of the links between stationary waves and regional climate and identified robust stationary wave responses to climate change, which include an increased zonal lengthscale in winter, a poleward shift of the wintertime circulation over the Pacific, a weakening of monsoonal circulations, and an overall weakening of stationary wave circulations, particularly their divergent component and quasi-stationary disturbances.

**Summary:**

Numerous factors influence Northern Hemisphere stationary waves, and mechanistic theories exist for only a few aspects of the stationary wave response to climate change. Idealized studies have proven useful for understanding the climate responses of particular atmospheric circulation features and should be a continued focus of future research.

**Electronic supplementary material:**

The online version of this article (10.1007/s40641-019-00147-6) contains supplementary material, which is available to authorized users.

## Introduction

Earth’s climate displays pronounced zonal (longitudinal) asymmetry. Principally responsible are atmospheric stationary waves, planetary-scale variations in the atmospheric circulation that are relatively stable on seasonal timescales. Stationary waves contribute, for example, to the relative dryness and coldness of Northern Hemisphere (NH) continents in midwinter [1, 2], the relative dryness of the Middle East, Mediterranean, and North Africa in summer [3, 4], the seasonal migration of precipitation in East Asia [5, 6], and the Pacific-Atlantic asymmetry in ocean freshwater forcing [7]. Changes in stationary waves with global warming therefore play an important role in determining the regional impacts of climate change. Uncertainties in the stationary wave response to climate change are a key source of uncertainty in future projections of regional climate [8].

Stationary waves arise from zonal asymmetries in topography, land-sea thermal contrast, atmospheric diabatic heating, and heat/momentum fluxes by synoptic (transient) eddies. The structure and amplitude of the stationary waves depend on the structure of the seasonally varying zonal-mean zonal winds (i.e., the jet stream). The strong seasonal cycle in diabatic heating [9], mechanical forcing by orography, and interactions between these forcings [10–12] produce a seasonal cycle in stationary waves. With stronger land-ocean temperature contrasts in winter, and stronger near-surface winds impinging on orographic slopes [9], extratropical stationary waves are strongest in winter; conversely, stronger low-latitude diabatic heating in summer produces a stronger subtropical stationary wave. In both seasons, changes in stationary waves can arise from changes in the zonally asymmetric forcing or from changes in the zonal-mean atmospheric state. Zonal asymmetries in diabatic heating and transient eddy fluxes are themselves dependent on the structure of the stationary waves [12–14] and can be considered feedbacks on the structure of stationary waves. Projected stationary wave changes are a complex superposition of changes due to these different mechanisms.

A range of modeling tools are used to predict and understand future changes in stationary waves. Comprehensive global climate models (GCMs) provide our best estimate of future changes in stationary waves and global climate. However, several studies have shown that the response of the extratropical atmospheric circulation to warming in comprehensive GCMs is sensitive to model parameters such as horizontal resolution and orographic gravity wave drag [15, 16], particularly through their influence on the background state and thus on the propagation of waves [17]. Furthermore, it can be difficult to gain mechanistic insight from these models, because the modeled atmospheric circulation changes are the net result of many different physical processes, and the interactions between stationary waves from different sources are generally nonlinear [10, 11, 18]. To separate the various influences on stationary waves, much of the classic literature has used stationary wave models, which solve linearized or weakly nonlinear versions of the equations of motion with a prescribed zonal-mean flow and prescribed diabatic tendencies [10–12, 19–28]. These models can accurately reproduce the climatological stationary waves given the specified zonal-mean flow and diabatic tendencies [22, 25], but the diabatic tendencies and (to a lesser extent) the zonal-mean flow are modified by stationary waves. A complete understanding of the mechanisms of stationary wave change therefore requires an understanding of the interactions between stationary waves, diabatic processes, and the mean state.

Atmospheric GCMs are used to study the interactions between stationary waves and diabatic processes; transient eddies are explicitly simulated, and the latent and radiative heating anomalies forced by stationary waves are allowed to feed back on the dynamics. To separate the various influences on stationary waves, a number of studies have specified simplified boundary conditions, such as localized surface heating or surface temperature anomalies [13, 29–32], simplified surface topography [14, 33–38], or simplified continental geometries [39–41]. Such idealized GCM simulations are useful for gaining physical understanding of different aspects of the stationary wave response to climate change, which in turn helps to determine which aspects of the comprehensive GCM projections are reliable. Here, our goal is to link mechanistic insights from idealized GCMs and stationary wave models with projected stationary wave changes in comprehensive GCMs.

This review also investigates how projected stationary wave changes depend on the metric used to measure them. Stationary waves comprise 3D structures in the time-mean zonally anomalous atmospheric circulation, including the zonal, meridional, and vertical winds. They can therefore be measured by longitudinal variations in any of these wind fields or by other representative variables such as the horizontal streamfunction, geopotential height, or sea-level pressure. The use of one variable over another can be motivated either by its dynamic importance or by its relevance for particular regional impacts.

In Stationary Wave Metrics, we review recent work identifying the stationary wave metrics relevant for particular climate impacts and introduce our analysis of projected stationary wave changes in comprehensive GCM simulations from the Coupled Model Intercomparison Project Phase 5 (CMIP5) [42]. We focus on tropospheric stationary waves in the NH midlatitudes (30°N–75°N) and connections with changes in the tropics (30°S–30°N). We separately discuss changes in NH Winter Stationary Waves (DJF) and NH Summer Stationary Waves (JJA). In each of these sections, we synthesize relevant understanding from theory, stationary wave models, and idealized GCM simulations and discuss how the projected changes relate to particular mechanisms. In Subseasonal Variability, we discuss projected changes in stationary wave variability on subseasonal timescales, such as that associated with so-called quasi-stationary waves, which has been highlighted as particularly important for climate impacts. We conclude with a Perspective, where we synthesize responses and mechanisms that are robust across models, discuss open questions, and make suggestions for future research.

## Stationary Wave Metrics

Changes in stationary waves are commonly measured in terms of a stationary wave horizontal streamfunction *ψ*^∗^, defined by

$$ {u}^{\ast }=-\frac{\partial {\psi}^{\ast }}{\partial y},\kern2em {v}^{\ast }=\frac{\partial {\psi}^{\ast }}{\partial x}. $$

Here, *u* and *v* are the zonal and meridional wind, respectively, and (·)^∗^ denotes the time-mean deviation from the zonal-mean, which we denote by [·]. In geostrophic balance, with constant Coriolis parameter *f*, atmospheric circulations can also be quantified in terms of the geopotential height *z* at constant pressure (*ψ* ≈ *gz*/*f*) or the pressure *p* at constant height (*ψ* ≈ *p*/*ρf*), e.g., sea-level pressure (SLP). These metrics capture only the rotational component of the flow. On planetary scales, the variation of *f* with latitude, *β* = *∂f*/*∂y*, gives rise to divergent flow and vertical motion through Sverdrup balance,

$$ f\frac{\partial \omega }{\partial p}\approx \beta v, $$

where *ω* is the vertical pressure velocity. Large-scale ascent can also arise from the rotational component of the stationary wave through frictional Ekman flow in the lower troposphere [7] or nonlinear wave interaction in the upper troposphere [43]. The rotational and divergent components of the stationary wave can be considered separately, as measured by *ψ*^∗^ and *ω*^∗^, respectively,^1^ or together, as measured by the horizontal winds *u*^∗^ and/or *v*^∗^. Additional dynamical variables such as potential vorticity, wave activity, and Plumb vectors [44] are useful for gaining insight into the mechanisms of stationary wave development and propagation.

**Fig. 1:**
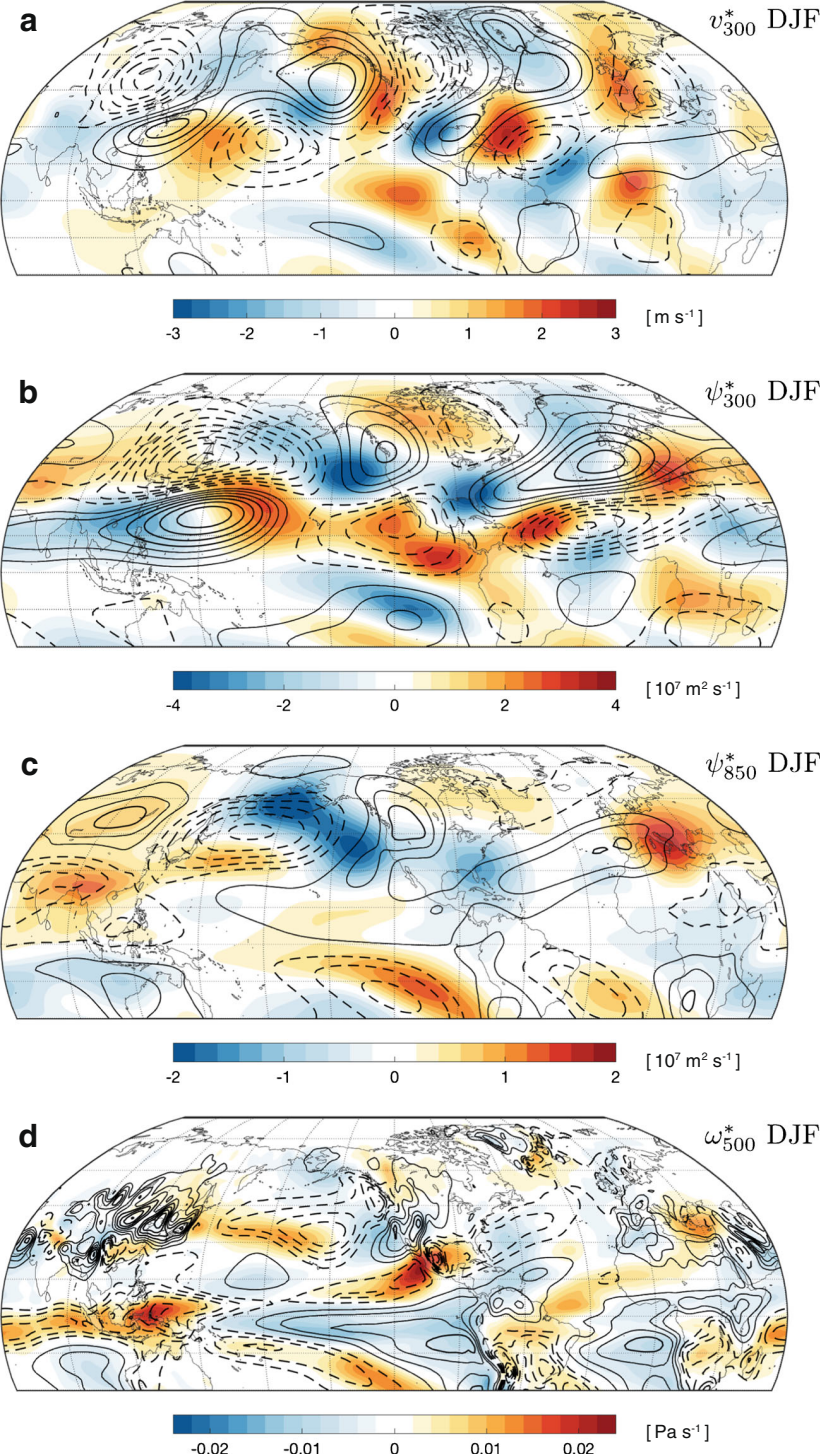
Climatology (1976–2005, contours) and climate change response (shading) of key measures of boreal winter (DJF) stationary waves, averaged over 39 CMIP5 models. Changes are differences between 2070–2099 in the RCP8.5 simulations and 1976–2005 in the historical simulations. ${\omega}_{500}^{\ast }$ is spatially filtered with a 1.5° Gaussian filter. The contour intervals for the black contours (climatologies) are **a** 2 m s^−1^, **b** 3 × 10^7^ m^2^ s^−1^, **c** 2 × 10^7^ m^2^ s^−1^, and **d** 0.012 Pa s^−1^. All map plots show latitudes between 30°S and 90°N and are centered at 120°W (which passes through California, Oregon, Washington, and British Columbia). See Supplementary Fig. S1 for additional stationary wave variables

**Fig. 2:**
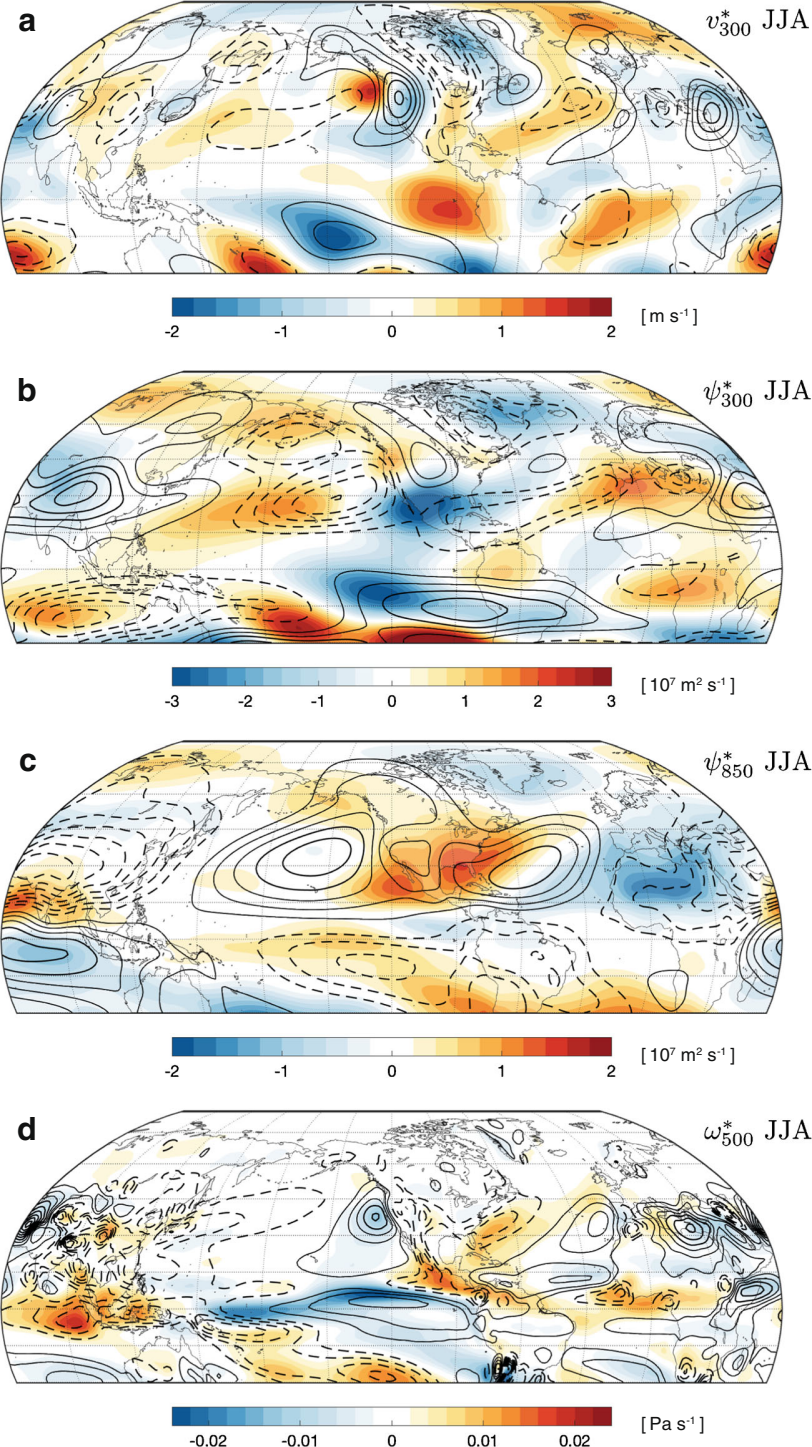
Same as Fig. 1 but for boreal summer (JJA). The contour intervals for the black contours (climatologies) are **a** 1.5 m s^−1^, **b** 2 × 10^7^ m^2^ s^−1^, **c** 2 × 10^7^ m^2^ s^−1^, and **d** 0.012 Pa s^−1^. See Supplementary Fig. S2 for additional stationary wave variables

Stationary waves exist throughout the atmospheric column, although we focus on the troposphere in this review. In winter, stationary waves are generally equivalent barotropic [12, 45], with the largest anomalies in the mid to upper troposphere but having the same sign throughout the troposphere (Fig. 1). In contrast, summer stationary waves are typically baroclinic, with opposite-signed anomalies in the upper and lower troposphere (Fig. 2), a consequence of forcing from diabatic heating within convective circulations [26, 46, 47]. It is therefore important to study anomalies in both the upper and lower troposphere (e.g., 300 hPa and 850 hPa) to understand barotropic and baroclinic stationary wave changes. Vertical velocities peak in the free troposphere for both barotropic and baroclinic circulations, and we therefore consider the vertical pressure velocity at 500 hPa as a representative vertical velocity. We also consider SLP and the geopotential height at 500 hPa (*z*_500_), which are frequently used to describe atmospheric circulation, particularly in the meteorology and climate variability literature.

One motivation for understanding how stationary wave changes compare across metrics is that different impacts are linked to different aspects of the stationary wave. For example, precipitation is largely determined by vertical motion whereas temperature anomalies are largely determined by meridional motion. Next, we review the recent literature focused on determining the relevant stationary wave metrics for particular impacts on regional temperatures, the hydrological cycle, and the stratospheric circulation.

### Metrics Relevant for the Hydrological Cycle

Transport of water vapor by atmospheric circulations controls regional variations in the hydrological cycle. The time-mean convergence of atmospheric water vapor transport sets the spatial pattern of precipitation minus evaporation (*P* − *E*). In the zonal mean, moisture-flux convergence in the inter-tropical convergence zone (ITCZ) and storm track lead to net precipitation (*P* − *E* > 0) whereas moisture-flux divergence in the subtropics leads to net evaporation (*P* − *E* < 0). Zonal variations in *P* − *E* are similarly set by zonal variations in moisture-flux convergence [7, 48, 49]. The hydrological cycle is expected to intensify under global warming due to the increase in atmospheric specific humidity, with wet regions getting wetter and dry regions getting drier [50–52]; however, changes in the atmospheric circulation modify this simple thermodynamic response. In the zonal mean, the influence of circulation changes is of second order, but for zonal anomalies, circulation changes are of leading order importance [53–55]

Stationary waves influence the hydrological cycle primarily through their influence on vertical motion. In particular, zonal variations in *P* − *E* (and its change) can be related to zonal variations in the divergent circulation [7, 36], with zonal variations in specific humidity of secondary importance. Stationary wave horizontal and vertical motions are linked through Sverdrup balance and Ekman pumping, which can be formalized in terms of a lower-tropospheric vorticity budget where boundary layer poleward/equatorward or cyclonic/anticyclonic motion leads to ascent/descent [7]. As a result of this relationship, large-scale precipitating systems, such as the Asian monsoon, can be characterized either by their rotational or by their divergent circulations.

There are also quantitative links between precipitation anomalies and vertical motion at various timescales [56–59]. Stationary waves can influence precipitation through their influence on time-mean vertical motions or through their influence on vertical velocity statistics, e.g., within storm tracks. For example, the localization of the NH storm tracks depends crucially on the interaction between stationary waves and transient eddies [13, 32, 60].

### Metrics Relevant for Regional Temperatures and Temperature Extremes

Stationary waves are also a primary influence on east-west variations in temperature. For example, they contribute to the warmth of Northern Europe relative to Eastern Canada [2, 61]. The zonal variance of temperature at midlatitudes can be thought of as arising from the meridional displacement *L* of time-mean streamlines in the presence of a zonal-mean meridional temperature gradient *∂*[*T*]/*∂y* [62–64]:

$$ \left[{T}^{\ast 2}\right]\sim {L}^2{\left(\frac{\partial \left[T\right]}{\partial y}\right)}^2. $$

Alternatively, the lengthscale *L* can be thought of as the product of a meridional velocity scale *V* and a timescale *τ* [14, 63, 65]:

$$ \left[{T}^{\ast 2}\right]\sim {\tau}^2{V}^2{\left(\frac{\partial \left[T\right]}{\partial y}\right)}^2, $$

where the timescale *τ* characterizes the processes acting to damp temperature anomalies set up by stationary wave circulations, such as transient eddy heat fluxes and radiative damping. The strength of meridional winds or the meridional displacement of streamlines are thus the relevant dynamic variables for changes in zonal temperature variance.

Similar arguments have been used to understand the temporal variance of temperature [63, 66]. In particular, some studies have suggested that periods with greater meridional displacement of the jet stream are associated with extreme temperature events in both winter [67–70] and summer [69–74], though it is still actively debated whether global warming and the associated Arctic amplification have an influence on the statistics of these events [75–79]. These studies highlight the importance of quasi-stationary waves, Rossby waves that persist for longer than a week but do not necessarily influence the long-term climatology. In Subseasonal Variability, we consider how quasi-stationary waves (and variability of stationary waves more generally) are projected to change in the future, focusing in particular on subseasonal variations in zonally anomalous meridional winds.

For both temporal and zonal variations in temperature, the influence of dynamics is generally small compared with the thermodynamic influence of changes in the lower-tropospheric meridional temperature gradient [14, 63, 80]. However, shifts in stationary wave circulations, particularly in the meridional winds, can lead to large regional temperature changes, especially at midlatitudes where the meridional temperature gradient is largest.

### Metrics Relevant for the Stratospheric Circulation

Planetary-scale waves are generally forced near the surface but propagate vertically into the stratosphere [81]. The associated wave breaking exerts an important influence on the stratospheric circulation (e.g., the polar vortex) [82–84]. While we focus on tropospheric stationary waves and their impacts in this review, we briefly discuss which aspects of stationary waves are relevant for the stratospheric circulation.

The strong stratification of the atmosphere above the tropopause traps all but the largest-scale waves in the troposphere [81], such that waves of zonal wavenumber *k* = 1 or 2 are the main influence on the stratosphere, and primarily at midlatitudes in winter. Changes in midlatitude stationary waves with wavenumbers 1–2 are therefore most relevant for understanding potential changes in stratospheric wave driving. For a more stratospheric focused look at how stationary waves are projected to change in the future, we refer the reader to Wang and Kushner [85], who show that a small increase in the wavenumber 1–2 tropospheric streamfunction at ∼ 60°N can lead to a substantial increase in stratospheric wave driving.

### Comparing Stationary Wave Changes Across Different Metrics in CMIP5

To assess the stationary wave response to global warming across different metrics, we analyze historical and RCP8.5 (business as usual) simulations from CMIP5 [42]. We use all 39 models for which monthly *u* and *v* fields are available (Table S1). We compute climatologies over the periods 1976–2005 in the historical simulations and 2070–2099 in the RCP8.5 simulations. We consider seasonal climatologies of *v*_300_, *v*_850_, *ψ*_300_, *ψ*_850_, *ω*_500_, *z*_500_, SLP, *u*_sfc_, and *u*(*p*). The streamfunction *ψ* is computed by solving for the inverse Laplacian of the vorticity in spherical coordinates. For all variables, the subscript refers to the pressure level in hectopascals. For months where the pressure level is below the surface at a grid point, we set velocities to zero and geopotential height to NaN (cf. [86]). All climatologies are interpolated to a common 1.5° analysis grid to compute multi-model means. Not all 39 models output geopotential height (*z*, 36 models) and vertical pressure velocity (*ω*, 38 models); the multi-model composites of these variables include all models for which the relevant variable is available (Table S1).

## Winter Stationary Waves

In boreal winter, the NH midlatitude stationary wave pattern has four dominant nodes. Their surface expression creates the Siberian high, the Aleutian low, the North American high, and the Icelandic low (Figs. 1 and S1). The stationary wave anomalies typically tilt westward with height, associated with vertical propagation into the upper troposphere and stratosphere [81]. Diabatic heating and mechanical orographic forcing both contribute to the maintenance of this midlatitude stationary wave [11, 12]. There are also stationary waves in the subtropical upper troposphere in winter, but they do not have a strong expression at the surface. The CMIP5 multi-model mean reproduces the observed winter stationary wave climatology remarkably well (Supplementary Fig. S3).

Before discussing the CMIP5 projections of future winter stationary wave changes, we consider how the classic literature suggests that stationary waves should change in a warming climate. Under warming scenarios, amplified low-level Arctic warming will act to weaken the lower-tropospheric equator-to-pole temperature gradient, *∂T*/*∂y*, while amplified tropical upper-tropospheric warming will strengthen *∂T*/*∂y* at upper levels [87]. These temperature gradient changes, along with the corresponding zonal wind changes (through thermal wind balance), can impact the amplitude, wavenumber, and phase of stationary waves.

From a dry dynamical perspective, the decrease in low-level *∂T*/*∂y* should lead to an increase in the amplitude of stationary waves. For extratropical stationary waves forced by diabatic heating, this heating is balanced primarily by meridional temperature advection [20]. If *∂T*/*∂y* weakens, then a larger stationary wave meridional wind *v*^∗^ is required to balance the diabatic heating. For stationary waves forced by orography, adiabatic cooling on the upslope side and warming on the downslope side are balanced by meridional advection, and, again, an increase in *v*^∗^ is required for balance in a climate with reduced *∂T*/*∂y* [12, 14, 20, 64, 88].

The stationary wave amplitude also depends on the low-level winds, which are projected to shift poleward with climate change [89, 90]. For thermally forced stationary waves, the amplitude is inversely proportional to the low-level zonal wind speed [24], while for orographically forced stationary waves, the amplitude is proportional to the speed of the wind impinging on the orography [20, 24], including the nonlinear modification of these winds by the stationary wave [18, 91, 92]. Stationary waves from orography at different latitudes have remarkably different amplitudes and propagation paths downstream [38]. As projected changes in low-level zonal winds are a function of latitude, stationary waves from orographic and thermal sources located at different latitudes will have different responses to climate change (e.g., [14]).

Regarding the wavenumber of stationary waves, linear theory of barotropic Rossby waves propagating on a zonal-mean flow away from sources (i.e., in the upper troposphere) connects the total wavenumber of stationary waves, *K*_S_, to the background flow [20, 93]:

$$ {K}_{\mathrm{S}}=\sqrt{k^2+{l}^2}=\sqrt{\frac{\beta -{\partial}^2\left[u\right]/\partial {y}^2}{\left[u\right]}}. $$

Here, [*u*] is the zonal-mean zonal wind and *k* and *l* are the zonal and meridional wavenumbers, respectively. For the same background flow and latitude (and therefore the same *K*_S_), this relation dictates that stationary waves with larger zonal wavenumber *k* must have a smaller meridional wavenumber *l* than those with smaller *k*.^2^ The stationary wavenumber *K*_S_ also serves as a refractive index for stationary waves, such that a local maxima in *K*_S_ (occurring in the zonal jets) can act as a waveguide, particularly for waves with larger zonal wavenumbers (*k* = 5–8), which are thus more likely to be meridionally trapped and circumglobal [20, 94, 95]. Larger waves, with smaller zonal wavenumbers (*k* < 5), are typically refracted equatorward, where they are absorbed or reflected at critical latitudes as [*u*] goes to zero [20, 94]. A decrease in *K*_S_ is projected for future climates [96], due to the projected increase in upper-level winds. If there is no corresponding change in meridional wavenumber, then the zonal wavenumber *k* of stationary waves must decrease (i.e., waves of larger zonal scale become stationary). This change in stationary wavenumber will also affect the propagation of stationary waves.

Changes in phase of stationary waves can largely be thought of as linked to changes in wavenumber, because the sources of stationary waves (mountain ranges, warm ocean regions, land-sea contrast) are to leading order fixed in space. For a fixed source of Rossby waves, a change in zonal wavenumber will lead to a change in the phase of the waves downstream away from the source [96]. Changes in stationary wave sources, such as from the reorganization of tropical convection [97] or from the poleward shift of the low-level jet [89, 90], may also lead to changes in the phase of stationary waves.

### Projected Winter Stationary Wave Response

Figure 1 shows the CMIP5 multi-model mean historical climatology (contours) and projected climate change response by end-of-century in RCP8.5 (shading) of boreal winter stationary waves. The magnitude of changes is on the order of 15–30% of the climatology. The first-order impact of climate change on the wintertime stationary waves is a shift in the phase [96]. This can be seen in the top three panels of Fig. 1 but is most apparent in the upper troposphere (panels a and b). This phase shift is associated with substantial changes in regional hydroclimates [49, 96], with important implications for water resources and flood events.

Comparison of Fig. 1 b and c elucidates the equivalent barotropic aspects of the climate change response: a Rossby wave train pattern from the Western Pacific to North America and a positive *ψ*^∗^ anomaly over Europe. In the lower troposphere, this manifests as a cyclonic anomaly over the North Pacific and an anticyclonic anomaly over Europe, with implications for the hydrological cycle over Western North America and the Mediterranean [98, 99]. This pattern is consistent with the barotropic response of 7 of 16 CMIP3 GCMs analyzed by Brandefelt and Körnich [100], who found that models with similar stationary wave response patterns tended to have similar zonal-mean circulation responses. This suggests that changes in the zonal-mean flow, rather than changes in diabatic heating and transient eddies, are a dominant control on the pattern of stationary wave changes, in agreement with other studies [28, 85]. This is because the particular spatial pattern of stationary wave anomalies depends on the ray propagation of the stationary waves [20] and is sensitive to small differences in the zonal-mean circulation.

**Fig. 3:**
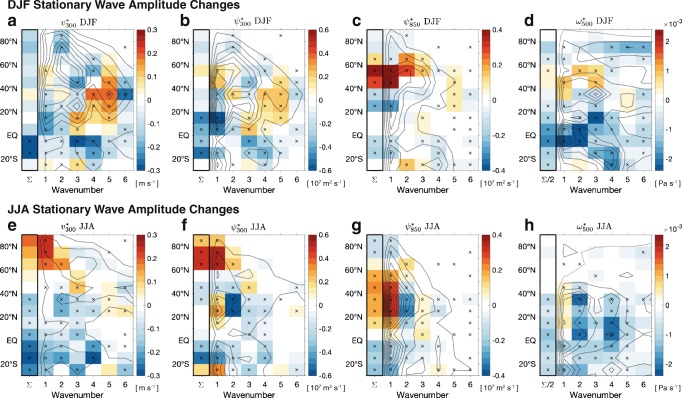
Climatology (1976–2005, contours) and climate change response (shading) of root mean square amplitude of (top) DJF and (bottom) JJA stationary waves (as a function of latitude and zonal wavenumber), as measured by several key stationary wave metrics. Climatological amplitudes are computed for each model separately in 2070–2099 and 1976–2005; then, the squared amplitude is averaged over the 39 CMIP5 models before taking the square root and then the difference. Stippling indicates where greater than 26 models agree on the sign of the change (a 0.01 significance level based on a binomial distribution). The left column in each panel shows the sum Σ over wavenumbers 1–6 (divided by a factor of 2 for ${\omega}_{500}^{\ast }$). The contour intervals for the dark gray contours (climatologies) are equal to the highest tick on the color bar for all panels. See Supplementary Fig. S6 for additional stationary wave variables

To average over small differences in propagation pathway, we investigate changes in stationary wave amplitude. Figure 3 shows the present-day amplitude (contours) and end-of-century changes (colors), calculated by Fourier transform analysis as a function of zonal wavenumber and latitude, for the metrics shown in Fig. 1. Given the large inter-model spread in the pattern of stationary wave response [100], we calculate amplitude changes for each model separately, before computing the multi-model mean. This identifies common changes in stationary wave amplitude across models, without requiring the background stationary wave, or the response, to be exactly in phase across models. The column on the left shows the total change summed over wavenumbers 1–6. Stippling shows a 0.01 significance level of agreement across models on the sign of the change. In the upper troposphere, the horizontal streamfunction and meridional wind show similar responses (Fig. 3a, b), with a broad decrease in stationary wave amplitude across many latitudes and wavenumbers, in particular for wavenumber 1 at mid-to-low latitudes. There is a slight increase in wavenumber 1 between 40°N and 60°N and a broader increase for wavenumbers 3–5 between the equator and 50°N. In the lower troposphere, however, the response appears rather different (Fig. 3c). There is a strong increase in wavenumbers 1–3 between 40°N and 70°N and only a weak signal of the midlatitude increase in higher wavenumbers that was seen at upper levels.

The first-order impact of warming on ${\omega}_{500}^{\ast }$ is a reduction in the amplitude of anomalies, both locally (Fig. 1d) and as a function of wavenumber (Fig. 3d), especially in the tropics and subtropics. The phase shift seen in the upper-tropospheric stationary wave circulation is not readily apparent in ${\omega}_{500}^{\ast }$, except for a northward and eastward shift of ascent within the North Pacific storm track that roughly follows changes in ${\psi}_{850}^{\ast }$. Similar to ${\psi}_{850}^{\ast }$, there is an increase in the amplitude of wavenumber 1–3 ${\omega}_{500}^{\ast }$anomalies between 40°N and 60°N.

**Fig. 4:**
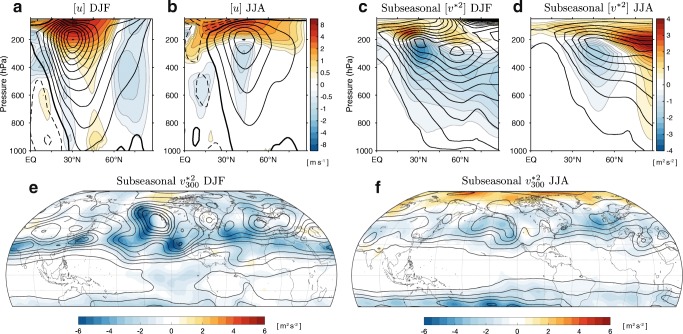
Climatology (contours) and climate change response (shading) of **a**, **b** the zonal-mean zonal wind, **c**, **d** the zonal-mean subseasonal variance of zonally anomalous meridional winds, and **e**, **f** the subseasonal variance of zonally anomalous meridional winds at 300 hPa in (**a**, **c**, **e**) DJF and (**b**, **d**, **f**) JJA, averaged over 39 CMIP5 models. Changes are differences between 2070–2099 in the RCP8.5 simulations and 1976–2005 in the historical simulations. Subseasonal variance is computed as the difference between the variance of monthly means and the variance of seasonal means. Contour intervals for the climatologies are **a**, **b** 4 m s^−1^; **c**, **d** 2 m^2^ s^−2^; and **e**, **f** 3 m^2^ s^−2^. The thick contour in **a** and **b** is the zero contour

Overall, this analysis shows a decreasing amplitude for most metrics, with increases only for specific latitudes and wavenumbers, in agreement with some [28, 96] but not all [100] previous GCM studies. This contrasts with the increase in amplitude predicted from dry dynamics with fixed diabatic heating, suggesting that diabatic processes are important in explaining this change. Note that while the stationary wave amplitude generally decreases in the troposphere, it increases in the stratosphere (Supplementary Fig. S5) consistent with an upward shift and strengthening of the zonal jet (Fig. 4a), as discussed in Wang and Kushner [85]. We will now consider how recent studies explain aspects of the projected stationary wave response highlighted here.

### Mechanisms of Winter Changes

The first-order effect of climate change on stationary waves is an eastward shift in phase. This phase shift is partly the result of an increase in the zonal wavelength of the stationary waves (a decrease in zonal wavenumber) of wavenumber 4–6 waves emanating from East Asia [96]. This decrease in wavenumber can be seen between 0° and 50°N in Fig. 3: a robust decrease in the amplitude of wavenumber 6 and corresponding increase in wavenumbers 4 and 5, particularly in the upper troposphere (panels a and b). Stationary wave theory links the wavelength of stationary waves to the speed of the background zonal wind [20, 93]; a lengthening of the wavelength in future climates is consistent with the simulated increase in upper-level zonal winds between 20°N and 60°N (Fig. 4a) and can be reproduced with a stationary wave model [96]. The reorganization of tropical convection with global warming may also contribute to the eastward shift in phase of midlatitude stationary waves and the pattern of stationary wave change more generally [97].

The poleward shift of the zonal-mean zonal jet in the mid-lower troposphere is a robust feature of the circulation response to warming in models and is coincident with a poleward shift of the storm track [89, 90, 101–103]. However, in the NH winter, this shift is largely from a signal in the North Pacific, as is apparent in the lower-tropospheric streamfunction and the near-surface zonal winds (Figs. 1c and S1) [89, 90, 104]. This is associated with a strengthening of the Aleutian low and an extension of the Pacific jet into California, which has been attributed to changes in tropical Pacific sea surface temperatures (SSTs) [105, 106]. In general, localized storm track and jet shifts are forced by a combination of stationary wave and transient eddy momentum fluxes [104]. However, idealized GCM simulations of zonally asymmetric storm tracks show that the latitude, strength, and zonal extent of storm tracks depend on interactions between stationary waves and transient eddies [13, 32, 60]. Therefore, while it is possible to characterize the momentum fluxes responsible for the zonal variation in the near-surface winds, a complete mechanistic understanding of these changes would require a theory for the two-way interaction of stationary waves and transient eddies.

Zonal asymmetry in the poleward jet shift contributes to the strong increase in the wavenumber 1 stationary wave between 40°N and 60°N (Figs. 3b–d). The increase in wavenumber 1–2 stationary waves at these latitudes may be important for its influence on the stratospheric circulation, as it suggests an increase in wave activity propagating into the stratosphere [85]. Arctic sea ice loss has also been suggested as an influence on tropospheric stationary waves, both directly and via the stratosphere [107–109]: sea ice loss can result in an increase in upward wave activity flux, producing a weakening of the stratospheric polar vortex; this may subsequently affect the wavenumber 1 and 2 tropospheric stationary waves through a downward control mechanism, although the relative importance of this “stratospheric bridge” has not been established.

The reduction in the magnitude of ${\omega}_{500}^{\ast }$ anomalies in the tropics (Figs. 1d and 3d) is consistent with a global slowdown of convective circulations with global warming [52, 56]. As vertical motion is coupled to horizontal wind changes through Sverdrup balance, the reduced amplitudes of the stationary wave meridional wind and streamfunction in the tropics (Fig. 3a–c) are likely linked to the slowdown of convective circulations. The mechanisms for the slowdown of convective circulations will be discussed in Mechanisms of Summer Changes, as this slowdown plays a broader role in the stationary wave changes in summer. Outside the tropics, changes in ${\omega}_{500}^{\ast }$, such as the reduction in subsidence in the eastern Pacific, are coupled to the horizontal wind changes through the lower-tropospheric vorticity balance (see, e.g., [7]).

There is little evidence for an overall increase in stationary wave amplitude with warming, as expected from dry dynamical arguments based on the reduced meridional temperature gradient ∂[T]/∂y [12, 64, 88]. In contrast, we see a reduced stationary wave amplitude across a range of latitudes, although this is not robust across models (Fig. 3a–c). The general weakening of stationary waves, particularly at wavenumbers *k* < 4, has been found in other studies [28, 96] but has not been explained. The role of moisture in reducing the effective slope of isentropes and therefore reducing the meridional wind anomaly needed to balance a given diabatic heating anomaly (as discussed by Wills and Schneider [14] in the context of orographically forced stationary waves) may play a role in this response. Alternatively, the weakening of stationary waves could result from reduced forcing from tropical convective circulations such as the Walker circulation [56, 97], which has been linked to the amplitude of winter stationary waves for the case of internal variability [110].

## Summer Stationary Waves

In boreal summer, stationary waves are primarily found in the NH tropics and subtropics, with the largest amplitudes between 15°N and 45°N (Figs. 2 and 3e–h). This is in contrast to winter, when stationary waves extend and peak further poleward (cf. Figs. 1 and 3a–d). The multi-model mean summer stationary wave climatology in CMIP5 models is in good agreement with the ERA-Interim reanalysis [111] (Supplementary Fig. S4); however, there remains poor agreement between models on present-day climatologies at the regional scale, due to their differing representations of key processes such as surface albedo [112], moist physics [113], and subgrid-scale topography [114].

An important characteristic of summer stationary circulations is their baroclinicity, consistent with large zonal anomalies in diabatic heating driving predominantly divergent flows in the tropics and subtropics (15°S to 45°N) [26, 47]. Over land, the strongest and most extensive baroclinic circulation is found over Asia and North Africa, with a low-level cyclone centered over northwestern India/Pakistan (Fig. 2c) and an upper-level anticyclone peaking over the Tibetan Plateau and the Persian Gulf (Fig. 2b). This circulation is associated with the South Asian monsoon, but its influence extends far beyond the region of diabatic forcing. Theoretical models, such as the Rossby gyre model of Gill [115], have shown that an upper-level anticyclone driven by monsoonal latent heating self-organizes to extend westward of its core [116, 117]. This leads to anomalous downwelling over the Zagros mountains and the eastern Mediterranean (Fig. 2d), which contribute to the aridity of those regions [4]. Beyond the monsoonal baroclinic circulation, there are many regions of shallow cyclonic circulation, known as heat lows, over subtropical arid zones such as the Sahara and Persian deserts. Despite their shallow vertical extent, these circulations interact strongly with neighboring monsoonal flows [118]. Their present-day variability and sensitivity to climate change remain poorly understood [119].

Baroclinic circulations also characterize the summer climate of the Pacific and Atlantic regions, with low-level anticyclones centered north of the Hawaiian Islands and east of the Caribbean Sea, respectively (Fig. 2c). These oceanic highs are regions of high surface pressure. Consistent with Sverdrup balance, regions of downwelling are found on the eastern flanks of oceanic anticyclones, corresponding to the semi-arid climate zones of coastal California and North Africa [3]. The Pacific and Atlantic anticyclones almost merge over North America, partially separated by a weaker monsoon system (the North American monsoon, Fig. 2c) [120].

### Projected Summer Stationary Wave Response

The strength of global convective mass fluxes is expected to weaken with global warming, as evaporation and latent heat release are energetically limited and cannot increase as fast as lower-tropospheric moisture content [52, 121]. To satisfy this global constraint, divergent stationary wave circulations are expected to weaken with global warming, because zonally anomalous overturning circulations make up a large fraction of the total convective mass flux [56]. Consistent with this global constraint, the mid-tropospheric vertical mass flux, ω^*^_500_, weakens over most regions (Fig. 2d). There is reduced ascent in the Maritime Continent, Central America, and the East Asian monsoon region and reduced subsidence in the eastern equatorial Pacific and the subtropical dry zones of coastal California, the eastern Mediterranean, and the Zagros mountains (Fig. 2d). Note that despite a global weakening of zonally anomalous overturning circulations, zonal asymmetries in precipitation (*P*) and hydrologic imbalance (*P* − *E*) are predicted to increase due to a strong increase in tropospheric specific humidity that overcompensates for the weakening of vertical motion [36, 49, 54, 122].

A weakening of the vertical mass flux does not necessarily imply weakening in other stationary wave metrics, such as horizontal streamfunctions or upper-level velocities. CMIP5 projections show a general tendency towards weakening of the horizontal stationary wave circulations in the tropics (Figs. 2 and 3), but the responses are more varied in the subtropics and midlatitudes. Across various metrics, changes are on the order of 15–30% of the climatology, similar to changes in winter. Climate models generally predict a weakening of the South Asian monsoon circulation with global warming, although quantitative agreement across models on the magnitude of this weakening is lacking and depends sensitively on how the circulation is diagnosed (e.g., [123]). The weakening of the South Asian monsoon is more apparent in the streamfunction changes than in the changes in vertical velocities (Fig. 2).

Models consistently suggest the appearance of two anomalous baroclinic structures with global warming, one over Central America and another over North Africa (Fig. 2). The former is characterized by a low-level anticyclonic anomaly centered over northern Mexico, which extends over large swathes of the southern US and into the Atlantic storm track (Fig. 2c). This baroclinic structure is associated with a weakening of the North American monsoon [124], an intensification of the anticyclonic ridge in the western US, and a weakening of the Atlantic storm track [102]. Stationary wave streamfunction changes over North Africa and the Mediterranean are characterized by a low-level cyclonic anomaly found over the Sahara and extending into the Sahel and southern Europe. Low-level anticyclonic tendencies over North America and cyclonic tendencies over North Africa strongly reinforce the dominant wavenumber 1 climatological pattern of ${\psi}_{850}^{\ast }$ in the 20°N to 50°N latitudinal band, while also weakening wavenumber 2 (Fig. 3g). This change in wavenumber is also apparent in the upper-tropospheric streamfunction ${\psi}_{300}^{\ast }$ (Fig. 3f).

Models also agree on the appearance of anomalous barotropic structures over the high-latitude oceanic regions with global warming. Specifically, anticyclonic tendencies are found over the North Pacific, while cyclonic tendencies are found over southern Greenland (Fig. 2b, c). These changes correspond to a northward expansion of the Pacific high and a strengthening of the Icelandic low; they are related to changes in the lower-tropospheric zonal winds (Supplementary Fig. S2). These changes manifest as a strong increase in the wavenumber 1 amplitude of ${\psi}_{300}^{\ast }$ and ${v}_{300}^{\ast }$ at latitudes greater than 60°N (Fig. 3).

### Mechanisms of Summer Changes

The weakening of divergent stationary circulations is broadly consistent with the global weakening of convective circulations, which is expected from global precipitation increasing at a slower rate than tropospheric moisture [52, 56, 57]. This constrains the gross vertical mass flux for all tropospheric circulations globally but does not necessarily hold for individual circulation features. A number of studies have therefore focused on local energetic constraints on divergent circulations. Knutson and Manabe [125] recognized that the cancelation of latent heating and adiabatic cooling within vertical motions precluded the need for increased circulation strength in response to increased latent heating in a warmer climate. Later studies have used the moist static energy (MSE) budget to account for the canceling effects of latent heating and adiabatic cooling on circulations, relating the strength of vertical motions to the net energy input to the atmospheric column and (inversely) to a measure of the MSE stratification called the gross moist stability [51, 126–130]. The gross moist stability generally increases with warming due to an increase in the depth of convection [129, 130], and this reduces the strength of circulations for a fixed energy input.

Changes in the large-scale summer stationary wave circulation have generally been understood through consideration of the land-sea MSE contrast or horizontal temperature and MSE gradients more generally [131, 132]. On large scales, MSE changes are relatively homogeneous across land and ocean, as expected from the weak temperature and moisture gradients in the tropical free troposphere [126, 133]; however, atmospheric circulations are sensitive to any small changes in MSE gradients [132]. One way of thinking about the circulation response to greenhouse gas forcing is as the residual of a fast direct effect on land surface temperatures and a slow indirect effect due to warming of SSTs [30, 134]. The direct effect increases the MSE thermal maximum and convective activity over land and thus strengthens stationary wave circulations (i.e., because the climatological MSE maximum is over land, and this change increases the zonal asymmetry of MSE; Supplementary Fig. S7). This is opposed by the indirect effect, where increasing SST increases the moisture content and MSE of the oceanic boundary layer, reducing the climatological land-sea MSE contrast that drives monsoonal flows. By strengthening convective activity over land (ocean), the direct (indirect) effect strengthens (weakens) zonal asymmetric circulations.

As the stationary wave response is a residual of opposing direct and indirect effects, even modest discrepancies between climate models in representing these processes can lead to a large spread in the predicted circulation change [30]. Feedbacks associated with large-scale ventilation of continents can further aggravate discrepancies between climate models, as changes in MSE over land are intrinsically tied to those of the surrounding ocean regions and may depend sensitively on physical parameterizations (e.g., [39, 114]). Most models show a robust drying (relative humidity decrease) and warming of the continental boundary layer as the climate warms [133]. This increases the land-to-ocean temperature (and MSE) contrast in the lower troposphere, especially over the western margins of dry subtropical continents (e.g., coastal California), where it has likely contributed to a strengthening of low-level stationary anticyclones over the Pacific and Atlantic basins in past decades and may further contribute to their strengthening as the climate warms [135]. Overall, there is a lack of agreement among climate models on the relative contributions of land drying and warming to stationary circulations changes with global warming. The CMIP5 multi-model mean projections show a weakening of convective activity over land in Asia and North America that leads to a weakening of the monsoonal stationary wave circulations (Fig. 2c, d).

In addition to changes in land-sea contrast, changes in SST patterns can drive changes in stationary circulations [55]. Indeed, a large fraction of the CMIP5 inter-model spread in the stationary wave response to global warming can be tied to model differences in SST changes over the subtropical oceans [136]. The influence of SST pattern changes on the tropospheric circulation cannot be accounted for by the direct-indirect effect compensation, since this mechanism relies on heterogeneous response of surface fluxes between land and ocean [137]. However, the influence of zonal asymmetries in near-surface SST and MSE on summer stationary circulations can be assessed from a general framework of planetary baroclinic Rossby waves, as shown in Levine and Boos [31]. Using this framework, a strengthening tendency of stationary circulations is predicted from an increase in the zonal contrast of MSE across the Pacific and Atlantic basins (Supplementary Fig S7) [122]. This increase in the zonal contrast of MSE results from the nonlinear dependence of near-surface moisture content on air temperature, which strongly amplifies MSE changes in the climatologically warm western boundary currents (Kuroshio and Gulf Stream) compared with the colder eastern parts of the Pacific and Atlantic ocean basins. In the ensemble-mean, however, this is overcompensated by the weakening tendency induced by the tropical-mean warming, which increases the gross moist stability and leads to an overall weakening of the zonally anomalous vertical mass flux.

## Subseasonal Variability

Variability in the amplitude and/or phase of stationary waves, such as that associated with quasi-stationary waves (QSWs), is thought to be associated with extreme midlatitude weather such as winter cold air outbreaks [67–70], summer heat waves [69–74], heavy precipitation [138–141], and drought [142–144]. QSWs are atmospheric Rossby waves which have a phase speed close to zero. QSWs that influence extreme weather are typically those with anomalously high amplitudes that persist for longer than synoptic timescales and are thus detectable in, for example, 15-day low-pass filtered data [70, 74] or monthly anomalies [69]. For the purpose of this review, we will consider QSWs to be any wave-like disturbance that persists for longer than two weeks but less than a season (i.e., subseasonal variability). This includes long-lived blocking events (though the blocking literature typically considers all events longer than 5 days, see, e.g., [145, 146]).

Quasi-stationary waves are a relatively new field of study, and there is not yet a clear consensus in the literature on how variability associated with QSWs will change in the future [67, 68, 71, 72, 75–79, 147, 148]. Some studies have suggested that Arctic amplified surface warming should lead to a slowdown of the zonal winds (through thermal wind balance) and an increase in the prevalence of large-amplitude quasi-stationary disturbances—a result of an increase in the stationary eddy wavenumber in winter [67, 68] or quasi-resonant amplification in summer [71–74, 148]. However, GCMs do not show a robust weakening of the zonal winds in response to Arctic amplification [77, 78, 96], partly due to the competing influences of tropical upper-tropospheric warming and the expansion of the troposphere. In fact, wintertime upper-tropospheric zonal winds are generally found to strengthen with warming in GCMs [96] (Fig. 4a, b). This leads to a decrease in the stationary wavenumber, rather than an increase (as discussed in Mechanisms of Winter Changes). Furthermore, Hassanzadeh et al. [147] showed that even when reduced meridional temperature gradient and zonal wind speed are imposed in an idealized GCM, the amplitude and meridional extent of *z*_500_ anomalies are reduced as a consequence of the reduction in mean meridional gradient of *z*_500_. In addition to the impact of these mean state changes on QSWs, anomalous land and ocean surface conditions may play a role in forcing QSW anomalies and must be considered in the context of climate change (see, e.g., [149]).

Much of the literature on QSWs has focused on the meridional extent of *z*_500_ excursions; however, in the context of climate change, the meridional extent metric has been shown to mix together mean state changes and wave changes [75, 77]. For this reason, it is preferable to consider the associated wind anomalies directly. We consider meridional winds because of their importance for temperature anomalies (see Metrics Relevant for Regional Temperatures and Temperature Extremes), consistent with some of the recent literature [71, 73, 76]. We analyze end-of-century (RCP8.5) changes in the subseasonal variance of zonally anomalous meridional winds, as simulated by 39 CMIP5 models. For simplicity, we compute subseasonal variance here as the variance of monthly means about each seasonal mean. Subseasonal meridional wind variance (averaged over all zonal lengthscales) extends throughout the midlatitude troposphere, with a local maximum between 200 and 300 hPa (black contours in Fig. 4 c and d). At 300 hPa, it is strongest over the eastern ocean basins, off the west coasts of North America and Europe (Fig. 4e, f). It is concentrated between nodes of the climatological ${v}_{300}^{\ast }$, particularly in winter, suggesting that this variability involves phase shifts of the climatological stationary wave.

The projected response of the subseasonal meridional wind variance to global warming is a weakening and upward shift in winter and a poleward and upward shift in summer (Fig. 4c, d). The increase in summertime meridional wind variance in the Arctic (Fig. 4d, f) has not been documented previously (to our knowledge) and may be relevant for future variability in sea ice. Zonal wavenumbers 6–8, which have been the predominant focus of the QSW literature, are responsible for most of the reduction in tropospheric meridional wind variance between 20°N and 50°N in both seasons (not shown). The weakening of midlatitude subseasonal meridional wind variance should contribute to a reduction in the subseasonal variance of temperature, though this is generally thought to be a secondary effect compared with changes in the mean meridional temperature gradient [63, 80]. This could be particularly relevant for changes in wintertime climate variability in southwest North American and the Persian Gulf and summertime climate variability in the British Isles and the US west coast, which all show strong reductions in meridional wind variance with warming (Fig. 4e, f).

Our analysis shows no evidence of increased variance of midlatitude (30°N–60°N) meridional winds in the CMIP5 simulations, as might be expected if QSWs increased in frequency and/or amplitude. More work needs to be done to reconcile these results with work suggesting that the meridional extent of QSW disturbances increases as a result of Arctic amplification [67, 68], although differences in timescales may play a role in this discrepancy. The projected decrease in meridional wind variance has been shown previously [76, 147] but has not yet been explained. In both seasons, the changes in subseasonal meridional wind variance are qualitatively similar to changes in the zonal variance of the climatological meridional wind (Supplementary Fig. S5), suggesting that similar mechanisms could play a role in both. We hypothesize that this change may be partially explained by the increased phase speed of Rossby waves in a strengthened zonal-mean flow (which means that anomalies are advected away before they can persist for a full month). The reduced lower-tropospheric meridional temperature gradient, increased lower-tropospheric MSE gradient, and any changes in the subseasonal variability of the zonal-mean winds could also influence the subseasonal variability of meridional winds.

Subseasonal variability in the zonally anomalous meridional winds, analyzed here, could result either from variation in the strength of stationary waves or from aliasing of synoptic variability onto monthly timescales. The structure of the climatological subseasonal variance suggests the former, because of strong variability in the Aleutian low and Icelandic low regions rather than extending throughout the storm tracks (Fig. 4e, f). However, more work needs to be done to distinguish these possibilities. One potential path forward is a decomposition of the standing wave and traveling wave components of the variance [150]. This could help to determine, for example, if these changes are linked to changes in storm track eddy kinetic energy, in which case an explanation can lean on the stronger theoretical underpinnings for how the energy of midlatitude transient eddies changes with global warming (see, e.g., [151]).

## Perspective

The response of Northern Hemisphere stationary waves to global warming is multifaceted, and we do not yet have a complete understanding of all the mechanisms responsible. Here, we summarize some of the stationary wave responses that are robust across models along with their mechanisms, highlight some open questions, and suggest future directions.

### Robust Responses

Robust responses are determined based on CMIP5 model agreement on the sign of change (e.g., stippling in Fig. 3), though in most cases there is still substantial model spread in the magnitude of change (not shown). Robust responses include:

1. Weakening of vertical winds south of 40°N in both seasons due to global energetic constraints on precipitation [52, 56] and an increase in gross moist stability [51, 128–130]; weakening of upper-tropospheric meridional winds south of 40°N in JJA that is likely coupled to the weakening of vertical winds through Sverdrup balance.
2. An increase in lengthscale of DJF stationary waves due to an increase in the upper-tropospheric zonal winds and a corresponding decrease in the stationary wavenumber *K*_S_ [96]. This decreases the amplitude of stationary waves for zonal wavenumber *k* ≥ 6 and increases the amplitude for *k* = 3–5, with particularly large impacts on upper-tropospheric meridional winds.
3. Baroclinic stationary wave responses over North America, North Africa, and South Asia in JJA that strengthen the wavenumber 1 streamfunction, weaken the wavenumber 2 streamfunction, and weaken the South Asian and North American monsoons. Although a complete mechanistic understanding is lacking, these changes are generally discussed in terms of changes in land-sea temperature/MSE contrasts and/or SST patterns (e.g., [30, 122, 132, 134]) and changes in static stability or gross moist stability [125, 128–130].
4. Weakening of the wavenumber 1 and 2 upper-tropospheric streamfunction and meridional winds at most latitudes in DJF. This weakening is likely linked to changes in diabatic heating, as could result from increased stationary wave latent heating [14] or the weakening of tropical SST gradients and tropical convective circulations [56, 97], but it has not been fully explained.
5. Barotropic anomalies over the North Pacific in both seasons, over the Mediterranean in DJF, and over the North Atlantic in JJA, due to zonal asymmetries in the poleward shift of the storm track and near-surface zonal winds. The mechanisms for the longitudinal and seasonal dependence of the poleward shift in the midlatitude circulation have been investigated by Simpson et al. [104], but a complete explanation still requires a better understanding of the two-way interaction between stationary waves and synoptic eddies (see, e.g., Kaspi and Schneider [13]).
6. Weakening of subseasonal meridional wind variability (such as that associated with quasi-stationary waves) at midlatitudes in both seasons and an increase in summertime variability in the Arctic. This reduction in variability has not yet been explained but can likely be attributed in part to the increase in upper-tropospheric zonal winds and the corresponding increase in the phase speed of Rossby waves.

### Open Questions and Path Forward

One of the most important issues highlighted by this review is the need to better understand slow variations in stationary waves (i.e., quasi-stationary wave activity). Variability in atmospheric circulations at subseasonal timescales is important for its impact on temperature and precipitation extremes but has received less mechanistic attention than either synoptic variability or the climatological atmospheric circulation. As such, the projected decrease in subseasonal meridional wind variance at midlatitudes and the increase in the summertime high-Arctic have been left largely unexplained. A better characterization of how these changes depend on lengthscale and timescale is needed in order to understand the connection of these changes with changes in the storm tracks and with changes in the climatological stationary waves. As midlatitude atmospheric variability is crucial in driving SST variability on longer timescales [152–155], future work should also investigate how these changes impact low-frequency atmosphere-ocean variability.

There remain open questions also on the response of the climatological stationary wave circulation to climate change. Research on stationary waves is at a point where the available tools (comprehensive and idealized GCMs, stationary wave models) provide a good representation of stationary waves, although the parameterization of subgridscale orography remains a challenge, with potential importance for projected stationary wave responses [16, 156]. However, a number of interesting stationary wave responses to climate change remain without complete mechanistic explanations. Establishing physical mechanisms for projected circulation changes can help to determine whether these projections are reliable. Future work should focus on particular robust responses, where models agree on the sign of change, and determine the sources of model spread in these responses. In this context it is beneficial to distinguish whether or not the model spread in a circulation response is linked to model spread in the global climate sensitivity and low cloud feedbacks [157–159]. Inter-model differences in the stationary wave response pattern are generally associated with differences in the zonal-mean atmospheric circulation [17, 100], which has a large influence on stationary wave propagation. This suggests that progress on constraining the zonal-mean circulation response will help constrain the stationary wave response pattern. Greater understanding is also required on the role of changes in wave propagation for projected change in stationary and quasi-stationary waves (e.g., [160]).

At midlatitudes, a number of the local circulation responses are related to zonal variation in the poleward shift of the atmospheric circulation. Simpson et al. [104] diagnosed the relative contributions of momentum fluxes by synoptic eddies and stationary waves to these local near-surface wind changes, but the mechanisms for the local storm track and stationary wave changes and their interactions remain to be explored. Research into how these processes respond to climate change could benefit from a hierarchical modeling approach [161, 162]. Two useful levels of the hierarchy are adding zonal asymmetries to an otherwise zonally symmetric climate in an idealized GCM (e.g., a storm track forced by a local heat source [13, 32]) or removing zonal asymmetries from a more realistic model (e.g., flattened mountain ranges [34, 38] or specified SST experiments [105, 163]). Climate change experiments within this model hierarchy will lead to insights into midlatitude stationary wave-transient eddy interactions and stationary wave responses more generally.

## Electronic Supplementary Material

ESM 1 (PDF 12484 kb)

## Footnotes

1 The divergent component of the flow can also be characterized by the velocity potential *Φ*, defined by $$ \left(\begin{array}{c}u\\ {}v\end{array}\right)=\nabla \varPhi $$.

2 Note that streamfunction anomalies with a larger zonal wavenumber have larger meridional wind anomalies, because *v*^∗^ = *∂ψ*^∗^/*∂x*, and streamfunction anomalies with a larger meridional wavenumber have larger zonal wind anomalies, because *u*^∗^ = − *∂ψ*^∗^/*∂y*.
