# Supplementary material for: Northern Hemisphere Stationary Waves in a Changing Climate
Source: Curr Clim Change Rep. 2019 Nov 21;5(4):372–89. doi: 10.1007/s40641-019-00147-6 (PMC6934250; doi:10.1007/s40641-019-00147-6)
Supplement: Supplementary file 1 — (PDF 12484 kb) [file 40641_2019_147_MOESM1_ESM.pdf]

Current Climate Change Reports

Supplementary Material for "Northern Hemisphere Stationary Waves in a Changing Climate"

Robert C. J. Wills · Rachel H. White · Xavier J. Levine

Contents:

- Supplementary Table S1
- Supplementary Figures S1-S7

Corresponding Author: Robert C. Jnglin Wills
E-mail: rcwills@uw.edu

Table S1 CMIP5 models used and variables used from each

Variables	Models
u, v, ω, z, MSE	ACCESS1-0, ACCESS1-3, bcc-csm1-1, bcc-csm1-1-m, BNU-ESM, CanESM2, CCSM4, CESM1-BGC, CESM1-CAM5, CNRM-CM5, CSIRO-Mk3-6-0, FGOALS-g2, FIO-ESM, GFDL-CM3, GFDL-ESM2G, GFDL-ESM2M, GISS-E2-H, GISS-E2-R, HadGEM2-AO, HadGEM2-ES, inmcm4, IPSL-CM5A-LR, IPSL-CM5A-MR, IPSL-CM5B-LR, MIROC5, MIROC-ESM, MIROC-ESM-CHEM, MRI-CGCM3, NorESM1-M, NorESM1-ME
u, v, ω, z	CMCC-CESM, CMCC-CM, CMCC-CMS, MPI-ESM-LR, MPI-ESM-MR
u, v, ω, MSE	GISS-E2-H-CC, GISS-E2-R-CC, MRI-ESM1
u, v, z, MSE	HadGEM2-CC

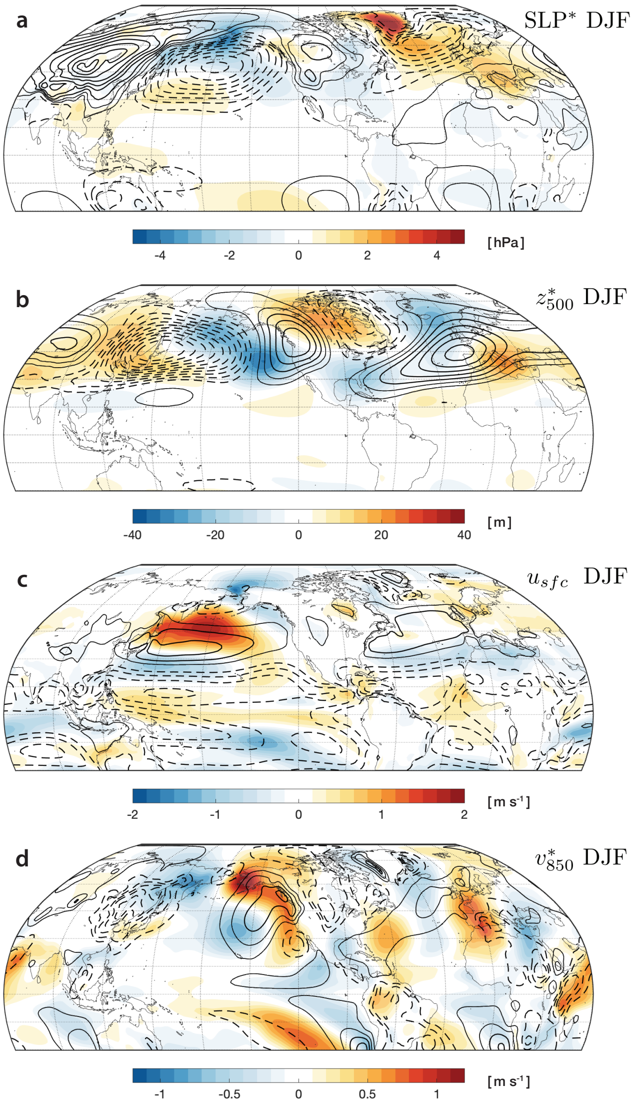

Fig. S1 Climatology (1976-2005, contours) and climate change response (shading) of key measures of Boreal winter (DJF) stationary waves, averaged over 39 CMIP5 models. Changes are differences between 2070-2099 in the RCP8.5 simulations and 1976-2005 in the historical simulations. v^*_{850} is spatially filtered with a 1.5° Gaussian filter. The contour intervals for the black contours (climatologies) are 2 hPa, 20 m, 2 m s^{-1}, 1.2 m s^{-1}, and 4×10^{-6} s^{-1}, from top to bottom.

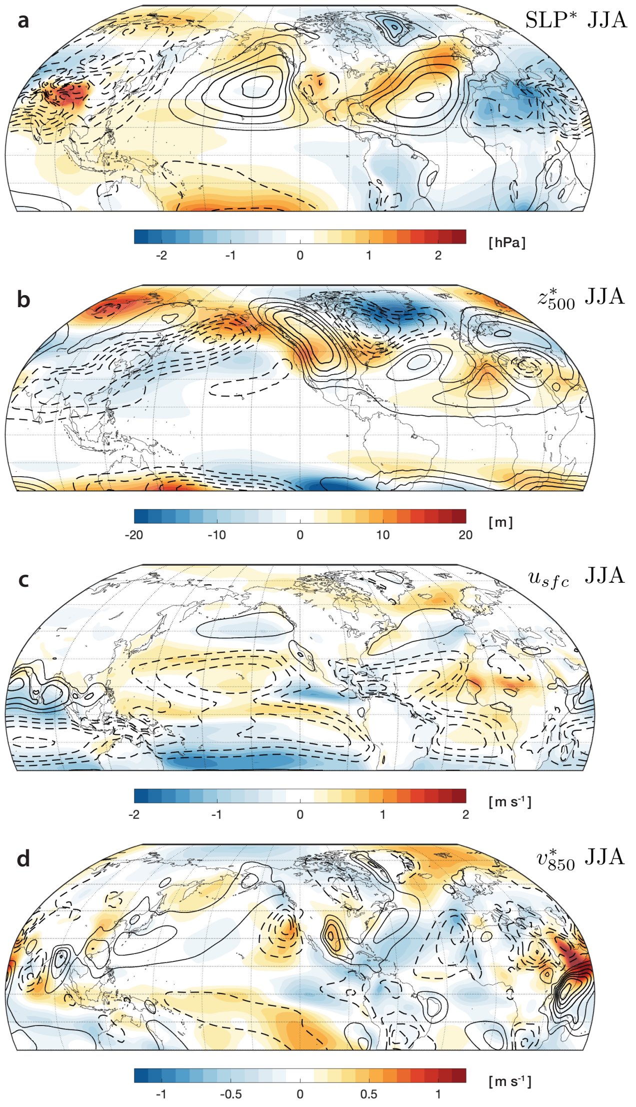

Fig. S2 Same as Fig. S1, but for Boreal summer (JJA). The contour intervals for the black contours (climatologies) are 2 hPa, 10 m, 2 m s^{-1}, 1.2 m s^{-1}, and 3×10^{-6} s^{-1}, from top to bottom.

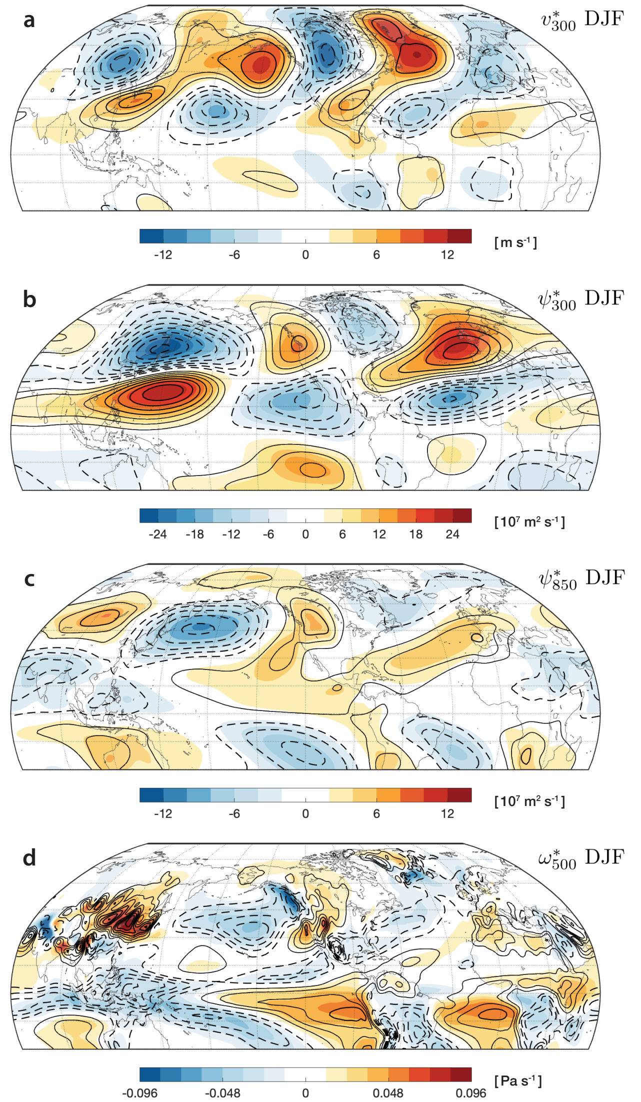

Fig. S3 Comparison of the DJF climatologies in CMIP5 (1976-2005, contours) and the ERA-Interim Reanalysis (1979-2012, shading). The ERA-Interim climatology for ω^*_{500} is shifted by a year to 1980-2013. Contour intervals are the same for the black contours and the shading, and are the same as the black contours in Fig. 1.

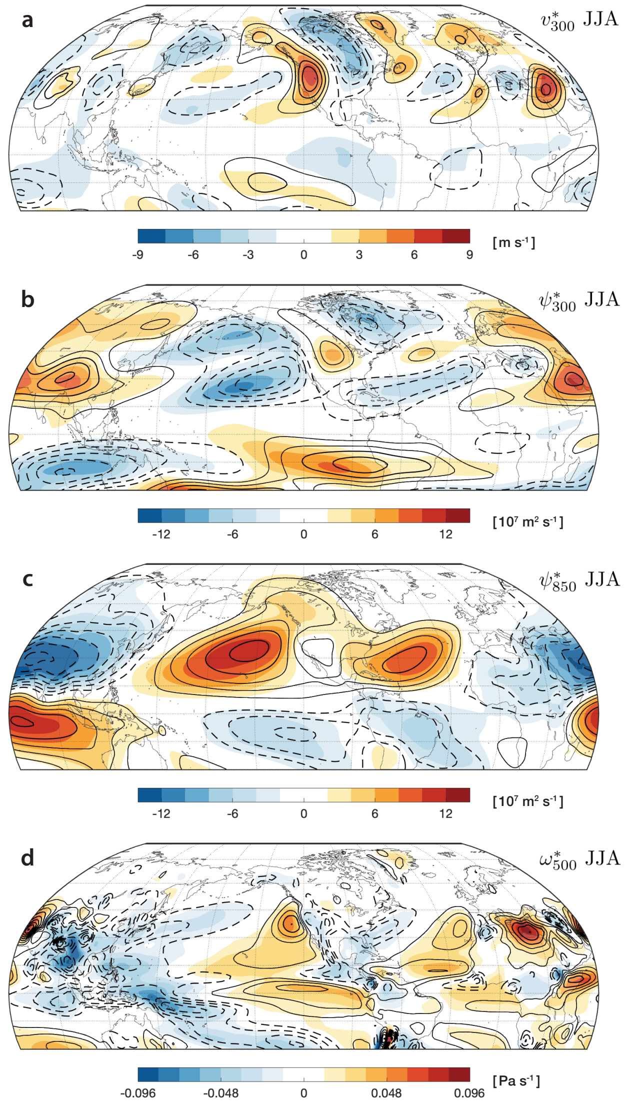

Fig. S4 Comparison of the JJA climatologies in CMIP5 (1976-2005, contours) and the ERA-Interim Reanalysis (1979-2012, shading). The ERA-Interim climatology for ω^*_{500} is shifted by a year to 1980-2013. Contour intervals are the same for the black contours and the shading, and are the same as the black contours in Fig. 2.

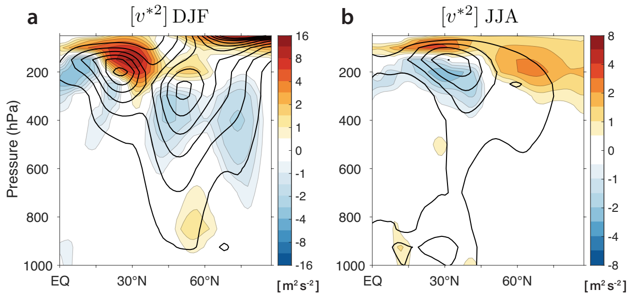

Fig. S5 Climatology (contours) and climate change response (shading) of zonal variance of the time-mean meridional wind in (a) DJF and (b) JJA. Contour intervals for the climatology are 5 m^2s^{-2} in (a) and 3 m^2s^{-2} in (b).

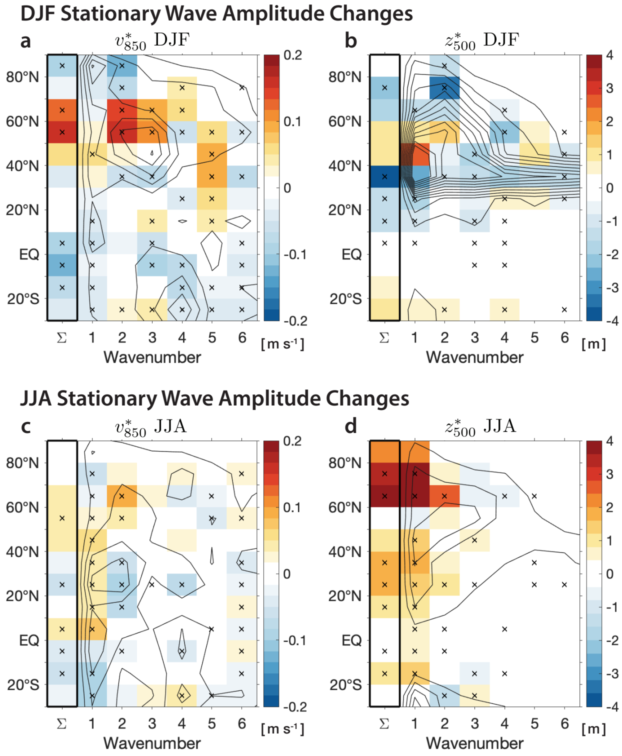

Fig. S6 Same as Fig. 3, but for additional stationary wave variables.

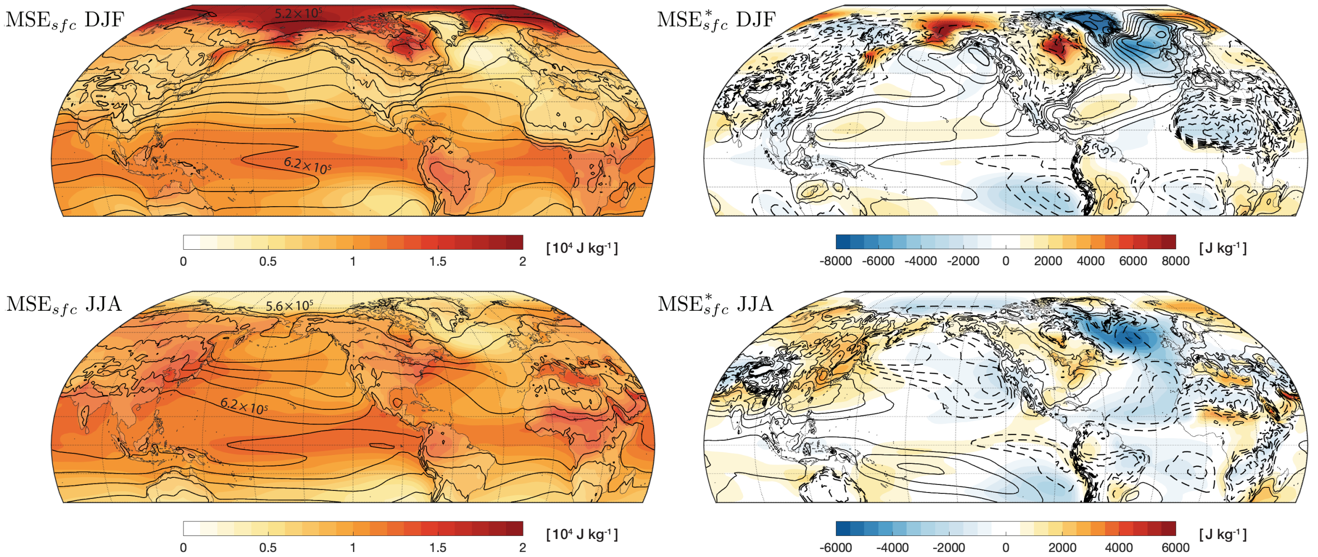

Fig. S7 Historical climatology (contours) and climate change response (shading) of near-surface moist static energy (MSE_{sfc}) and its zonally anomalous component (MSE^*_{sfc}) in DJF and JJA. Changes are differences between 2070-2099 in the RCP8.5 simulations and 1976-2005 in the historical simulations. Only the 34 models that output the needed surface temperature and surface specific humidity are included in this multi-model composite (Table S1). The contour interval for the climatologies are 0.2×10^5 J kg^{-1} for MSE_{sfc} and 3000 J kg^{-1} for MSE^*_{sfc}.
